# Role of prophylactic HIPEC in non-metastatic, serosa-invasive gastric cancer: a literature review

**DOI:** 10.1515/pp-2022-0104

**Published:** 2022-07-04

**Authors:** Aditya R. Kunte, Aamir M. Parray, Manish S. Bhandare, Sohan Lal Solanki

**Affiliations:** Department of Surgical Oncology, GI & HPB Surgery, Tata Memorial Hospital, Homi Bhabha National Institute, Mumbai, India; Department of Anaesthesiology, Critical Care & Pain, Tata Memorial Hospital, Homi Bhabha National Institute, Mumbai, India

**Keywords:** hyperthermic intraperitoneal chemotherapy, peritoneal carcinomatosis, recurrence, stomach cancer

## Abstract

The role of prophylactic hyperthermic intraperitoneal chemotherapy (p-HIPEC) in serosa invasive gastric cancers without gross or microscopic peritoneal disease, to reduce the rate of peritoneal relapse is an area of ongoing research. Although p-HIPEC is effective in reducing the rate of peritoneal relapse and improving disease free and overall survival with or without adjuvant chemotherapy, when added to curative surgery in locally advanced, non-metastatic gastric cancers, the available literature is at best, heterogeneous, centre-specific and skewed. Apart from that, variations in the systemic therapy used, and the presence of the associated nodal disease further complicate this picture. To evaluate the role of p-HIPEC the PubMed, Cochrane central register of clinical trials, and the American Society of Clinical Oncology (ASCO) meeting library were searched with the search terms, “gastric”, “cancer”, “hyperthermic”, “intraperitoneal”, “chemotherapy”, prophylactic”, “HIPEC” in various combinations, and a critical review of the available evidence was done. Although p-HIPEC is a promising therapy in the management of locally advanced gastric cancers, the current evidence is insufficient to recommend its inclusion into routine clinical practice. Future research should be directed towards identification of the appropriate patient subset and towards redefining its role with current peri-operative systemic therapies.

## Introduction

The peritoneal failure after curative gastric resection is the most common mode of recurrence accounting for 36–45% of all the recurrences [1], [2], [3]. Given the dismal prognosis associated with peritoneal recurrence (median survival of 2.7 months and 1-, 3-, and 5-year survival rates of 61.0, 19.9, and 9.9%, respectively), its prevention remains a key concern during management of gastric cancer [1]. To select the patients for peritoneum directed therapies at the time of curative gastric cancer resection for non-metastatic disease, identification of the high-risk group becomes essential. Higher incidence of peritoneal recurrences have been reported with the diffuse–mixed histological type, infiltration of the serosa, lymph node involvement, tumour size, grade, higher AJCC tumour stage, perineural invasion, and lack of adjuvant chemotherapy [1, 3]. The 5-year cumulative risk of peritoneal recurrence in the diffuse-mixed type of gastric cancer has been reported to be 12% in the absence of serosal invasion, and 69% in the presence of serosal invasion [3]. Koga et al. highlighted the impact of intra-peritoneal free cancer cells (IFCCs) and serosal invasion on the survival of patients with gastric cancer, reporting 5-year survival of 85% in patients lacking both, 40% in patients with only serosal invasion, and 13% in patients with both risk factors; with peritoneal recurrence being the most common pattern of recurrence in patients with either of these two risk factors [4]. Boku et al. reported the 5-year survival rates of patients with and without serosal invasion to be 47.1 and 75.9% respectively [5]. With this background, prophylactic hyperthermic intraperitoneal chemotherapy (p-HIPEC) has been utilized as a promising option to minimize the peritoneal relapses in patients with high risk features. We have critically reviewed the present literature to evaluate the role of p-HIPEC in locally advanced non-metastatic gastric cancers.

## Materials and methods

### Literature search strategy

An electronic literature search was conducted using the databases of ‘PubMed’, ‘Cochrane Central Register of Controlled Trials’, ‘American Society of Clinical Oncology (ASCO) meeting library’. The period for the search was from 1980 to October 2021. The search terms included, “gastric”, “cancer”, “hyperthermic”, “intraperitoneal”, “chemotherapy”, “prophylactic”, “HIPEC” and their synonyms in various combinations. The search also included all MeSH terms. The extracted articles were further reviewed in a step-wise manner for identification of relevant studies. The titles and abstracts were inspected independently by two authors (A.R.K. and M.B.). In addition, a search for ongoing studies using the same search terms was done using the clinical trial registries, ‘ClinicalTrials.gov’, and the ‘Chinese Clinical Trials registry (ChiCTR)’.

### Study selection criteria

Only articles published in English were included for review. Only articles regarding prophylactic HIPEC in gastric cancer were included. Articles regarding application of HIPEC in metastatic gastric cancer were excluded. Articles regarding gastric cancer patients with positive peritoneal cytology or gross peritoneal disease were excluded. Articles without an abstract or full-text were excluded. Only original research articles were included for analysis. Meta-analyses and review articles were excluded.

### Literature search results

**Figure 1:**
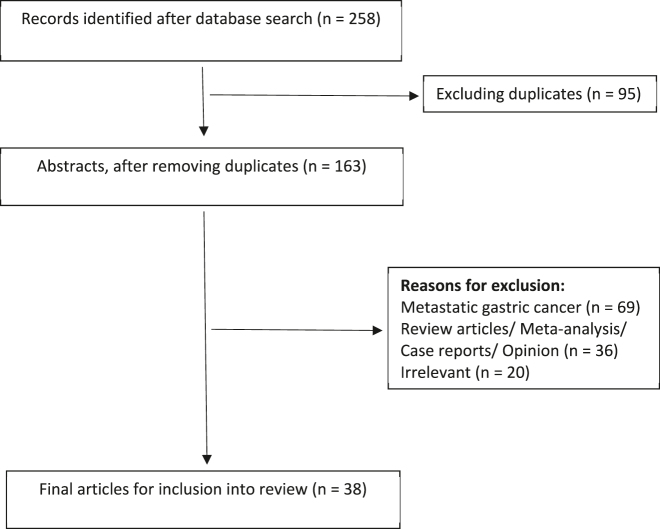
Flowchart for literature search.

A total of 258 articles were identified after the initial literature search. Initial review included screening of article titles for relevance and identifying duplicates. A further screening of abstracts identified articles for full text review. Full text assessment identified 26 original articles and 12 ongoing studies regarding prophylactic HIPEC in gastric cancer for inclusion into the final review article. The literature search flowchart is shown in Figure 1.

### Ethical approval

This study was a review of evidence available in literature. No human participants were involved in this study, hence this study was deemed exempt from an ethical review by the institutional Ethics Committee.

## Rationale for prophylactic HIPEC (p-HIPEC)

Peritoneal carcinomatosis usually originates from IFCCs, which can seed the peritoneum after spontaneous exfoliation from the primary tumour [4, 6, 7]. The IFCC positivity rate increases with increasing T-stage and serosal invasion of the primary tumour, as well as with the involvement of regional lymph nodes [8, 9]. In addition to that radical surgery intended for cure also inadvertently contributes to dissemination of tumor cells into the peritoneal cavity, as cancer cells are released into the peritoneum from transected lymphatic channels, tissues at the narrow margin of resection, and tumor-contaminated blood lost in the surgical field from the cancer specimen [6, 10, 11]. In addition to that, the presence of the plasma-peritoneal barrier accounts for the poor penetration of intravenous chemotherapy into the peritoneum limiting the impact of systemic chemotherapy. However, this very property can be leveraged to our advantage, as reported by Morgan et al. [12], who demonstrated a 1,116 fold higher peak peritoneal concentration than peak plasma concentrations of intra-peritoneally administered Gemcitabine, there-by enabling chemotherapeutic agents to effectively eradicate IFCCs, micro-metastases, and tumor nodules while limiting systemic adverse effects. An additional advantage of intra-peritoneal administration of chemotherapeutics, albeit theoretical, is that drugs administered into the peritoneal cavity are ultimately absorbed through the portal vein into the liver and may thus have an anti-tumoral effect on liver micro-metastases as well [13]. In addition to that hyperthermia increases the efficacy of intra-peritoneal chemotherapy, not only by potentiating the cytotoxic effect of the chemotherapeutic agents, but by direct tumoricidal effects as well. Hyperthermia causes ischemic necrosis of tumour tissue by causing microvascular embolism in the tumour tissue microenvironment. It also disturbs cancer cell homeostasis and energy metabolism by activating lysosomes, interfering with DNA, RNA, and protein synthesis, as well as disrupting cell membrane proteins, thereby causing direct cancer cell lysis. The synergistic effect of hyperthermia with chemotherapy is enacted via increasing the penetration of chemotherapeutics into tumour nodules, increasing the drug uptake in tumour cells and increasing the chemosensitivity of neoplastic cells [14], [15], [16].

## Current evidence

The earliest evidence regarding the role of p-HIPEC in improving outcomes and reducing rates of peritoneal recurrences in locally advanced, serosa-invasive gastric cancer, without evidence of peritoneal disease came from two Japanese studies, reported by Koga et al. (1988) [17] and Kaibara et al. (1989) [18]. Koga et al. reported results from two studies, the first a retrospective study with a historical control, and the second a randomized controlled trial. Prophylactic HIPEC administered with mitomycin C (MMC) for 60 min at 40–42 C in histologically confirmed serosa invasive gastric cancer without macroscopic peritoneal deposits demonstrated an improvement in OS (3-year OS 73.7 vs. 52.7%) as well as a decrease in the rate of peritoneal recurrences (36 vs. 50%), with respect to a historical control from the same institute [17]. Though not statistically significant, these results provided the basis for the first RCT for prophylactic HIPEC in non-metastatic, serosa invasive gastric cancer, which included a total of 47 patients and demonstrated a trend towards improved OS with prophylactic HIPEC. Kaibara et al. was also able to demonstrate improved 5-year OS rates (71.5 vs. 59.7%) with 50–60 min of prophylactic HIPEC with MMC [18]. These studies paved way for multiple Western and eastern studies which are discussed below in detail.

### The Western experience

Majority of the Western experience of p-HIPEC for locally advanced gastric cancer comes from Europe. The evidence is predominantly in the form of retrospective non-comparative studies, with only two non-randomized retrospective comparative studies, and one RCT from Belarus [19], [20], [21], [22], [23], [24], [25], [26], [27]. The details of the retrospective non-comparative studies have been summarised in Table 1.

**Table 1:** Retrospective non-comparative studies for p-HIPEC.

Study	Study type	n	Stage	Surgery	HIPEC	Systemic therapy	Outcomes
Roover et al. (2006) [19]	Retrospective	16	Stage IB-II-4	TG + D2-All Splenectomy (1), DPS (3), Transverse colectomy (3), left hepatectomy (1), limited peritonectomy (3)	MMC 15 mg/m^2^ 20–90 min (median 73 m) 42–43 °C Open technique	Adjuvant chemo-5 Adjuvant CTRT-1	5-year OS-72%
			Stage IIIA-IV-12				
Scaringi et al. (2008) [20]	Retrospective	11	pT3, pT4	TG + D1.5-D2	MMC 120 mg + Cis 200 mg/m^2^ 90–120 min 41–43 °C Open technique	No	Median OS 23.4 m 2 year recurrence rate 36% Median time to recurrence- 18.5 m
Graziosi et al. (2013) [21]	Retrospective	11	T4, peritoneal cytology +ve	TG + D2/D2+	MMC + Cisplatin 60 min	No	Median OS 29.6 m Median DFS 20 m
Saladino et al. (2014) [22]	Retrospective	12	pT3/pT4-N2 Peritoneal cytology −ve	TG + D2 ± splenectomy	Cisplatin 25 mg/m^2^/L MMC 3.3 mg/m^2^/L 60 min 41–43 °C Closed technique	Yes	Median OS 24 m 8.3% peritoneal recurrence
Privalov et al. (2017) [23]	Retrospective	6	Serosal invasion + Poorly differentiated diffuse-mixed histology	TG/DG + D2	Cisplatin 200 mg Closed technique	No	RFS 14 and 23 m
Yarema et al. (2019) [24]	Retrospective	37	pT4a/pT4b N0/N+	TG D0/D1-35% D1+/D2-65%	Cisplatin + MMC/Oxaliplatin/Cisplatin + doxorubicin 30–90 min 41–43 min Closed technique	Adjuvant chemo in 21%	Median OS-34 m Median DFS- 28 m 1-year OS- 91.7% 1-year DFS-

Yarema et al. [24] administered p-HIPEC with Cisplatin and MMC to 37 patients of serosa-invasive (cT4-N0/N+) gastric cancers without evidence of peritoneal metastases, and reported median overall survival (OS) and disease free survival (DFS) of 34 and 28 months respectively; and one-year OS and DFS rates of 91.7 and 82.3% respectively. However, 35% of patients underwent a less than D2 lymphadenectomy, and only 21% received systemic chemotherapy. These results demonstrate clearly the survival benefit of p-HIPEC; however, a substantial proportion of these patients underwent inadequate surgery or did not receive systemic chemotherapy, and hence the clinical applicability of these results remains limited.

Diniz et al. [26] included clinically staged non-metastatic cT3N+ and cT4 diffuse-type adenocarcinoma of the gastric body and antrum with negative peritoneal cytology into a p-HIPEC protocol in addition to standard peri-operative systemic chemotherapy with cisplatin, epirubicin or taxane based regimens. They failed to demonstrate a survival benefit by addition of prophylactic HIPEC with 5-year OS and DFS of HIPEC vs. non-HIPEC groups reported as 59.5 vs. 68.7%, and 49.5 vs. 65.8%, respectively. However, these results are heavily influenced by a case selection bias as the patient cohort receiving HIPEC were selected specifically for having a poor disease biology and a high risk for peritoneal recurrence. In light of this bias, the lack of a significant difference in the outcomes of these populations may well be a testament to the efficacy of p-HIPEC. Interestingly, both groups had similar rates of peritoneal failure, but the HIPEC group had a higher incidence of distant nodal recurrence, thus emphasizing the continued risk of distant failure despite addition of HIPEC and the continued need for effective systemic therapy.

Reutovich et al. [27] reported the only RCT from the Western hemisphere in which gastric cancers with histologically documented serosal invasion without peritoneal metastases were randomized to HIPEC and surgery only groups. None of the patients received systemic chemotherapy. HIPEC was administered with cisplatin and doxorubicin. Median PFS and 3-year PFS rates were significantly higher in the HIPEC group; 28 vs. 13 months and 47 vs. 27% respectively. Rates of peritoneal recurrence were also significantly lower in the HIPEC group (12.8 vs. 27.6%). A major criticism of this trial, however, is the omission of systemic chemotherapy in locally advanced gastric cancer, limiting the applicability of the results of this trial to patients who have received pre-operative or peri-operative systemic chemotherapy.

### The Asian experience

Compared to the evidence from the European countries, the evidence available in support of p-HIPEC for locally advanced gastric cancer from their Asian counterparts is quite robust, comprising of nine non-randomized case-control studies (Table 2) and nine randomized clinical trials (Table 3), with the majority of early evidence originating from Japan, and more recent evidence originating from Chinese institutions [17, 18, 28], [29], [30], [31], [32], [33], [34], [35], [36], [37], [38], [39], [40], [41], [42].

**Table 2:** Non-randomized comparative studies for p-HIPEC.

Study	Study type	n	Stage	HIPEC	Systemic therapy	Outcomes
Coccolini et al. (2016) [25]	Retrospective case-control	HIPEC-6 Surgery alone-28	pT3/pT4 Peritoneal cytology −ve	Cisplatin 100 mg/m^2^ + Paclitaxel 75 mg/m^2^ 90 min 40–41 °C	NACT	Median OS-34.6 m 27–28 m; p=0.04 Median DFS-34.5 m vs. 21–27 m; p=0.03
Diniz et al. (2020) [26]	Retrospective case control	HIPEC-28 Control-241	IIA-III	MMC 38 mg/m^2^/Cisplatin/Oxaliplatin-200 mg/m^2^/Cisplatin 30 mg/2 + docetaxel 30 mg/m^2^ 90 min 41–42 °C	Perioperative chemotherapy FOLFOX/XELOX/EOX/ECF/CF/DCF	5-year OS-59.5 vs. 68.7 m; p=0.45 5-year DFS-49.5 vs. 65.8 m; p=0.06 Recurrence-46 vs. 22%; p=0.001 Peritoneal recurrence-53 vs. 46%; p=0.002 Distant nodal recurrence-30 vs. 8%
Koga et al. (1988) [17]	Retrospective case control	HIPEC-59 Control-78	Macroscopic serosa +ve No macroscopic peritoneal deposits	MMC 64–100 mg 60 min 40–42 °C CHPP-M technique	No	3-year OS-73 vs. 52%; p=0.04 5-year OS 63 vs. 43% Peritoneal recurrence-36 vs. 50% Hematogenous recurrence-18 vs. 40%
Yonemura et al. (1995) [28]	Retrospective case-control	HIPEC-79 Control-81	Macroscopic serosal invasion, no peritoneal deposits	MMC-30 mg CDDP-300 mg 60 min 40–42 °C CHPP technique	Oral UFT 400 mg/day-adjuvant-2–3 weeks	No OS benefit in microscopic serosa invasion negative tumours Significant 5-year OS benefit in histologically confirmed serosa invasive disease; p=0.016 Significant OS benefit in stage IV disease
Hirose et al. (1999) [29]	Retrospective case-control	HIPEC-15 Control-40	Macroscopic serosal invasion, no peritoneal deposits	100 mg of CDDP, 20 mg of MMC, and 100 mg of etoposide 60 min Open technique	Weekly adjuvant 5FU + MMC × 3 weeks	3 year OS-48 vs. 28%; p=0.0142 5-year OS-39 vs. 17% Median OS-33 vs. 22 m Peritoneal recurrence-26 vs. 45%
Kim et al. (2001) [30]	Prospective non-randomized	HIPEC-51 Control-52	pT3/pT4	MMC 40 mg IHCP-2 h 42 °C	Adjuvant 5FU/5FU + MMC × 6 in 38 pts (HIPEC) and 43 pts (control)	5-year OS 32 vs. 27% (NS) 5-year OS excluding stage IV-58 vs. 44%; p=0.03
Kunisaki et al. (2002) [31]	Prospective non-randomized	HIPEC-45 Control-79	Macroscopic serosal invasion, no peritoneal deposits, peritoneal cytology −ve	150 mg cisplatin, 15 mg mitomycin C, and 150 mg etoposide 40 min 42–43 °C Open technique	Adjuvant 5FU + Mtx + CDDP in 26 (HIPEC and 39(control) patients	No significant 5-year OS benefit Peritoneal recurrence- 11 vs. 21%
Murata et al. (2016) [32]	Retrospective	186 total	pT3-4	MMC + CDDP ± 5-FU Temperature: 42–43 °C Time: 30 min	No	3-year OS-94 vs. 59%; p<0.0001 5-year OS-86 vs. 53% Significant benefit for hepatic (p=0.033) and peritoneal recurrence free survival (p=0.005)
Kang et al. (2013) [33]	Retrospective	HIPEC-29 Control-83	pT3/pT4	Cisplatin (30 mg/L), mitomycin (10 mg/L), and etoposide (20 mg/L) 60 min 41–43 °C	Adjuvant chemotherapy in 21–22% in either group	5-year OS-43 vs. 10%, p=0.029 Mean OS-34 vs. 21 m
Zhu et al. (2020) [34]	Retrospective	22-CHIP 21-Control	Stage IIA-IIIC	Cisplatin 75 mg 30 min 42 °C EPIC	*Cis*-5FU 2 adjuvant cycles minimum	Median DFS-36.5 vs. 24.5 m; p=0.044 Median OS-NR vs. 33 m; p=0.037
Cheng et al. (2021) [35]	Retrospective	99-HIPEC 71-Control	Locally advanced	–	Adjuvant chemo	3-year OS-48 vs. 41.4%; 5-year OS-22.9 vs. 14%

**Table 3:** Randomized controlled trials for p-HIPEC.

Study	n	Stage	HIPEC	Systemic therapy	Outcomes	p-Value
Koga et al. (1988) [17]	HIPEC-32 Control-28	cT3/cT4	CHPP: MMC 8–10 mg/L, total dose 64–100 mg Temperature: in 44–45 °C, out 40–42 °C Time: 50–60 min	No	30-month OS-83 vs. 67% (NS)	<0.04^a^
Kaibara et al. (1989) [18]	HIPEC-42 Control-40	cT3/cT4	CHPP: MMC 10 mg/L, total dose 20 mg. Temperature: in 44–45 °C, out 40–42 °C. Time: 50–60 min	No	5-year OS 71 vs. 59% Peritoneal recurrence 11.9 vs. 20%	–
Hamazoe et al. (1994) [36]	HIPEC-42 Control-40	cT3/cT4	10 mg/L mitomycin C in 2 L of perfusate, 40–45 °C, 50–60 min	No	5-year OS 64 vs. 52% (NS) Peritoneal recurrence 39 vs. 59% Hematogenous recurrence 17 vs. 18%	0.24
Ikeguchi et al. (1995) [37]	HIPEC-78 Control-96	cT3	^a^	MMC + oral UFT	5-year OS 66 vs. 44% (NS) in N+ subgroup Median peritoneal RFS 30 vs. 23 m	0.084
Fujimoto et al. (1999) [38]	HIPEC-71 Control-70	cT3/cT4	10 mg/L mitomycin С in 3–4 L of perfusate, 43–44 °C, 120 min	Adjuvant immunochemotherapy-Sizofiran (SPG)	4-year OS 76 vs. 58% Significantly less peritoneal recurrence	0.036^a^
Reutovich et al. (2019) [27]	HIPEC-76 Control-78	pT4 Histologically confirmed serosa invasive disease	Cisplatin 50 mg/m^2^ + doxorubicin 50 mg/m^2^ 60 min 42 °C Open technique	No	3-year PFS-47 vs. 27% Median PFS-28 vs. 13 m Peritoneal recurrence rate- 12 vs. 28%	0.0024^a^ <0.001^a^
Yonemura et al. (2001) [39]	HIPEC-48 Control-47	cT2-cT4	30 mg mitomycin С + 300 mg CDDP in 6–8 L of perfusate, 42–43 °C, 60 min	Adjuvant	5-year OS-61 vs. 42% Significant benefit in serosal invasion and N+	–
Beeharry et al. (2019) [40]	HIPEC-40 Control-40	cT3-cT4	Cisplatin 50 mg 41–43 °C 60 min Open technique	Adjuvant XELOX	3-year DFS 93 vs. 65% Peritoneal recurrence rate 3 vs. 25%	0.0054^a^ <0.05^a^
Xie et al. (2020) [41]	HIPEC-51 Control-62	cT4	Cisplatin 50 mg/L 42–43 °C 60 min Open	Adjuvant XELOX/SOX	3-year DFS-63 vs. 60% 3-year OS- 68 vs. 66%	0.037^a^ 0.044^a^
Fan et al. (2021) [42]	HIPEC-33 Control-17	cT3–cT4	Cisplatin 50 mg 42–43 °C 60 min	Adjuvant SOX	3-year OS 87.9 vs. 100%	0.142

^a^Statistically significant.

One of the earlier landmark non-randomized studies which was published by Yonemura et al. [28] provided early evidence for dual agent HIPEC with MMC and Cisplatin. Outcomes of patients with serosa invasive non-metastatic gastric cancer undergoing a curative resection and HIPEC were compared to those of controls undergoing curative resection alone, without HIPEC. All patients received adjuvant chemotherapy with oral UFT. A statistically significant 5-year OS benefit was observed in the HIPEC group (50 vs. 30%) with comparable post-operative mortality of 3.8 vs. 2.5%. This study was significant because it provided important early evidence of the safety and efficacy of p-HIPEC in serosa-invasive gastric cancer in addition to adjuvant oral chemotherapy.

Other retrospective Asian studies have reported their experience of p-HIPEC with MMC based regimens, in addition to 5-FU/MMC based systemic therapies. These studies all demonstrate a reduction in rate of peritoneal recurrence, however, a few studies seem to suggest that survival benefit may be restricted in patients with microscopically confirmed serosal invasion. Peritoneal lavage cytology was also not done routinely, thus raising a possibility of stage migration significantly affecting these results.

Among the RCTs, before year 2001, evidence for efficacy of Mitomycin-C as the principal chemotherapeutic agent in HIPEC, is provided by the Japanese trials [17, 18, 36], [37], [38], [39], whereas the Chinese trials [40], [41], [42] provide more recent evidence for the use of single agent Cisplatin HIPEC in conjunction with multi-agent systemic chemotherapy.

Of the six Japanese trials, five trials have utilized single agent MMC, of which one trial, reported by Ikeguchi et al. [37], administers adjuvant therapy with oral UFT in addition to HIPEC. Locally advanced serosa invasive gastric cancer without macroscopic peritoneal disease undergoing a curative gastrectomy were randomized to HIPEC and non-HIPEC arms. HIPEC was administered with MMC for 60 min. Oral UFT was administered as adjuvant chemotherapy in all patients. Mean DFS and 5-year OS rates of HIPEC vs. non-HIPEC groups were reported as 30 vs. 24 months, and 66 vs. 44% respectively.

Yonemura et al. [39] compared the outcomes of surgery alone, chemo-hyperthermic peritoneal perfusion (CHPP), and chemo-normothermic peritoneal perfusion (CNPP) in cT2-cT4 gastric cancer. Intra-peritoneal chemotherapy was given with Cisplatin and MMC and adjuvant chemotherapy was given to all patients after surgery. A significant OS benefit was observed only in those patients with serosal invasion or those with N+ disease receiving CHPP with 5-year OS 61% vs. 42%. This trial provided evidence for the efficacy of not just intra-peritoneal chemotherapy, but also highlighted that the benefit of HIPEC in gastric cancers is limited to a subset of patients with microscopic serosal invasion or nodal metastases. However, these findings were from a subset analysis and the trial was not powered to assess the efficacy of HIPEC in this subset.

Three recent Chinese RCTs, all published after 2019, provide evidence for efficacy of single agent p-HIPEC with cisplatin in addition to adjuvant systemic chemotherapy with XELOX/SOX. All three trials included patients with macroscopic serosal invasion. The results of these RCTs are conflicting, as the trials reported by Beeharry et al. [40] and Xie et al. [41] showed a significant DFS benefit by addition of p-HIPEC, with significantly reduced rates of peritoneal recurrence, however, Fan et al. [42], did not report any OS or RFS benefit by addition of p-HIPEC. p-HIPEC, did not increase the post-operative morbidity in any of the trials.

In summary, the Asian experience provides considerable evidence regarding the efficacy of p-HIPEC in preventing peritoneal recurrences and in prolonging survival. However, inadequate staging, stage migration, and heterogenous systemic chemotherapy regimens plague the earlier studies, whereas the newer trials show conflicting results, with only a few trials providing conclusive evidence of benefit.

### Ongoing trials for prophylactic HIPEC

The ongoing clinical trials for p-HIPEC have been summarized in Table 4 [43], [44], [45], [46], [47], [48], [49], [50], [51], [52], [53], [54]. A few key trials are discussed below.

**Table 4:** Ongoing clinical trials for p-HIPEC.

Trial registration	Study description	Study participants	Study arms	Primary endpoint	Current status
NCT01882933 GASTRICHIP [43]	Phase III RCT Multicentre Location: France n=367	T3, T4 and/or N+ and/or with positive peritoneal cytology	Arm A: Curative gastrectomy with D1–D2 lymph node dissection + HIPEC with oxaliplatin Arm B: Curative gastrectomy with D1–D2 lymph node dissection	5-year OS	Estimated completion- 2026
ChiCTR1900024552 DRAGON-II [44]	Phase III RCT Single centre Location: China	cT4 gastric cancer Peritoneal cytology negative or positive	Arm A: Combined Neoadjuvant chemotherapy + Neoadjuvant Laparoscopic HIPEC + D2 gastrectomy Arm B: D2 gastrectomy alone	Progression free survival	Not yet recruiting
NCT04597294 CHIMERA trial [45]	Phase III RCT Multicentre Location: Poland n=600	cT3/cT4a/N0-3b.	Arm A: Perioperative FLOT 4 + surgery + pre-operative HIPEC (Irinotecan) Arm B: Perioperative FLOT 4 + surgery	6-month peritoneal recurrence rate	
NCT02528110 [46]	Phase II RCT Location: Wuhan, China n=100	T3–T4 stage	Arm A: D2 gastrectomy + adjuvant SOX/XELOX Arm B: D2 gastrectomy + adjuvant SOX/XELOX + HIPEC (paclitaxel + 5FU)	5-year OS	Completed recruitment
NCT02356276 [47]	Phase III RCT Multicentre Location: China n=584	cT3/cT4 gastric adenocarcinoma	Arm A: D2 gastrectomy + adjuvant SOX/XELOX Arm B: D2 gastrectomy + SOX/XELOX + postoperative HIPEC (paclitaxel)	5-year OS	Recruiting
NCT02381847 [48]	Phase III RCT Single centre Location: Jiangsu, China n=60	cT3/cT4 gastric and GEJ adenocarcinoma	Arm A: D2 gastrectomy + adjuvant SOX/XELOX Arm B: D2 gastrectomy + SOX/XELOX + intra-operative HIPEC (cisplatin)	2-year OS	Was recruiting Current status unknown
NCT03917173 [49]	Phase III RCT Multicentre Location: Italy n=240	c T3-T4 N0-N+ gastric adenocarcinoma +ve peritoneal cytology	Arm A: D2 gastrectomy + adjuvant chemotherapy Arm B: D2 gastrectomy + adjuvant chemotherapy + intra-operative HIPEC (MMC + cisplatin)	3-year DFS	Recruiting
NCT02396498 [50]	Phase III RCT Single centre Location: China n=270	Stage III gastric adenocarcinoma	Arm A: D2 gastrectomy + adjuvant IV Cisplatin + oral S-1 Arm B: D2 gastrectomy + post-operative HIPEC (cisplatin) + adjuvant oral S-1	5-year OS	Was recruiting Current status unknown
NCT02240524 [51]	A phase III RCT Single centre Location: Guangzhou, China n=582	cT4 gastric adenocarcinoma	Arm A: D2 gastrectomy + adjuvant XELOX Arm B: D2 gastrectomy + adjuvant XELOX + intra-operative and postoperative HIPEC (paclitaxel)	5-year OS	Was recruiting Current status unknown
NCT02960061 [52]	Phase III RCT Single centre Location: Guangzhou, China n=640	cT3-cT4/N1-N3/M0 gastric adenocarcinoma	Arm A: Neoadjuvant chemotherapy (mDOF) + D2 gastrectomy + adjuvant chemotherapy (XELOX/SOX) Arm B: Neoadjuvant chemotherapy (mDOF) + D2 gastrectomy + adjuvant chemotherapy (XELOX/SOX) + post-operative HIPEC (paclitaxel)	5-year OS	Was recruiting Current status unknown
NCT02205008 [53]	Phase III RCT Multicentre Location: Korea n=230	Locally advanced gastric cancer Radiological suspicion of serosal invasion	Arm A: D2 gastrectomy + adjuvant S-1 Arm B: D2 gastrectomy + adjuvant S-1 + EPIC (MMC)	5-year OS	Was recruiting Current status unknown
NCT02269904 [54]	Phase II RCT Multicentre Location: China n=120	Stage III gastric adenocarcinoma	Arm A: D2 gastrectomy + adjuvant XELOX Arm B: D2 gastrectomy + adjuvant XELOX + intra-abdominal 5FU implants	3-year DFS	Was recruiting Current status unknown

#### GASTRICHIP (NCT01882933) [43]

This is an ongoing trial of p-HIPEC in locally advanced gastric cancer after D2 resection. This trial includes T3-4 gastric cancer with serosal invasion and/or positive lymph nodes and/or positive peritoneal cytology. They are randomized into two treatment arms of surgery only and surgery + HIPEC. HIPEC is given with oxaliplatin. Primary outcome measure is 5-year OS. Expected date of study completion is 2026.

#### DRAGON II trial (ChiCTR1900024552) [44]

This is the first RCT investigating the safety and efficacy of neoadjuvant laparoscopic HIPEC (NLHIPEC) in gastric cancer. The Dragon II regimen comprises of one cycle of NLHIPEC for 60 min at 43 ± 0.5 °C with 80 mg/m^2^ of Paclitaxel followed by three cycles of NAC with SOX regimen and after assessment, standard R0 D2 gastrectomy with intraoperative HIPEC followed by five cycles of SOX regimen chemotherapy. The control group will undergo standard R0 D2 followed by eight cycles of AC with oxaliplatin with S-1 (SOX) regimen. All potential subjects will undergo a diagnostic laparoscopy and patients with macroscopic peritoneal disease will be excluded. Study end points are 5-year PFS, 5-year OS, peritoneal metastasis rate (PMR), and morbidity rate. The study aims to recruit 326 patients of locally advanced gastric cancer which will be randomized into the two treatment arms in a 1:1 ratio.

#### CHIMERA trial (NCT04597294) [45]

This is a Polish trial designed to evaluate the efficacy and safety of peri-operative FLOT4 with HIPEC in locally advanced gastric cancers at high risk for peritoneal metastasis. HIPEC is administered with irinotecan. The trial aims to include 600 patients with a primary end-point of 6-month peritoneal recurrence rate. It is estimated to be completed by 2026.

## Discussion

The first step in the evolution of the treatment of gastric cancers was the development of surgical procedures to achieve a radical lymphadenectomy, given the high rates of lymph node involvement even in early gastric cancers. Rates of nodal metastases have been reported to range from 10 to 50% for T1 tumors, with rates as high as 80–90% for T2 to T4a tumors [55]. The evidence for the efficacy of radical lymphadenectomy came from the Dutch Gastric Cancer trial which showed significantly lower gastric cancer related deaths and locoregional recurrences with a D2 lymphadenectomy [56]. However, even with the addition of radical lymphadenectomy, median survivals for stage III tumors ranged between 15 and 29 months, with 5-year survivals between 17 and 40% [57]. The predominant pattern of failure was a hematogenous recurrence (54%), followed by peritoneal (43%), local (22%), and nodal (12%) recurrence [58].

The next advance in the management of gastric cancer was the advent adjuvant and peri-operative systemic chemotherapy as demonstrated in landmark studies such as the ACTS-GC, CLASSIC, MAGIC, and the FLOT-4-AIO trial [59], [60], [61], [62]. Two large meta-analyses confirmed that maximal OS benefit, PFS prolongation, and significant reduction in rates of distant recurrences could be achieved by adding peri-operative chemotherapy to curative surgery in locally advanced gastric cancer [63, 64]. However, despite significant improvements in OS, recurrence rates continued to remain as high as 26% even after multi-modality treatment, with extremely poor survival after recurrence [65]. The most common site of failure was the peritoneum, with up to 49% recurrences occurring in the peritoneum, as opposed to only 20% in the liver [65].

These figures point to the imminent obstacle in the management of gastric cancers, which is peritoneal recurrence, for which p-HIPEC seems to be the most promising modality. Most of the early literature examines p-HIPEC in a stand-alone setting in combination with curative surgery, or with the addition of older systemic chemotherapy regimens, rather than a part of multimodal treatment protocols with highly effective modern chemotherapy regimens, which have now become the standard of care. The evidence does show that p-HIPEC is effective in reducing rates of peritoneal recurrences, but the magnitude of improvement in OS and PFS is similar to what was seen in the initial experience with adjuvant systemic chemotherapy when it was compared to surgery alone. Given that rates of hematological and peritoneal recurrences are nearly equal after surgery alone, it stands to reason that p-HIPEC alone with curative surgery would produce a benefit akin to that of systemic therapy and surgery, a fact reflected well in the available evidence.

The strength of the existing evidence in favor of p-HIPEC is insufficient to persuade one to abandon the current standard of peri-operative chemotherapy in favor of p-HIPEC. Although the existing literature suggests that serosal invasion is a risk factor for peritoneal recurrence and presence of higher nodal burden is a risk factor for hematogenous recurrence [66], few reports also confirm peritoneum as the commonest relapse site in high nodal stage disease such as pN3 stage [67]. Given the fact that as high as 80–90% of T3/T4a tumors are associated with nodal disease, the risk of distant hematogenous recurrences in this group of gastric cancers remains substantial, despite the increased risk of peritoneal disease, thus further weakening any argument for omitting peri-operative chemotherapy in this sub-group.

This brings us to the most glaring lacuna in the available evidence for p-HIPEC thus far, which lacks robust, large-scale studies to evaluate the potential benefit of p-HIPEC in addition to modern peri-operative chemotherapy regimens, namely, FLOT. Due credit must be given to the evidence emerging from Asian institutions, where p-HIPEC has shown to produce a significant DFS benefit when added to curative surgery with adjuvant systemic chemotherapy. However, the newer Asian RCTs are phase II trials, and these findings can only be extrapolated to the Asian populations where adjuvant chemotherapy comprises of oral S-1 as a single agent or in combination with platin chemotherapy. Moreover, due to the pharmacogenomic differences in Western patients, the maximum tolerated dose of S-1 with cisplatin is lower than that in Asian patients, hence prohibiting the wider application of these results around the globe [68].

## Conclusions

In conclusion, although individual, small-scale studies from the Western countries show a trend towards better outcomes with prophylactic HIPEC, high quality evidence demonstrating the benefit of prophylactic HIPEC in addition to established systemic chemotherapy regimens in locally advanced, serosa invasive gastric cancer is lacking. The Asian experience, on the other hand provides robust evidence for the use of mitomycin, mitomycin-cisplatin, and cisplatin along-with systemic adjuvant therapy for the prevention of peritoneal recurrence in the form of well conducted randomized clinical trials. However, level 1 evidence in the form of randomized clinical trials for assessing the efficacy of adding p-HIPEC to the current standard of peri-operative chemotherapy regimens such as FLOT is still lacking, and some of the ongoing studies will hopefully yield results that would propel us across yet another threshold in the management of locally advanced gastric cancers.
